# A new era for research publication: Will Open Access become the norm?

**DOI:** 10.1111/jdi.13174

**Published:** 2019-11-20

**Authors:** Nigishi Hotta

**Affiliations:** ^1^ Department of Internal Medicine Chubu Rosai Hospital Japan Organization of Occupational Health and Safety Nagoya Japan

## Abstract

The progress of JDI travels in optimistic paces with optimistic persons. A remarkable rise in Impact Factor was observed after the introduction of Open Access. It seems that there is a positive relationship between online usage and Impact Factor.

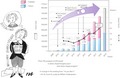

Scientists and publishers worldwide are closely following developments in the European Open Access landscape following the announcement that "top European research funders announce ‘Plan S’ to make all scientific works free to read”^1^, ^2^. “No science should be locked behind paywalls”: this powerful declaration was released by 11 agencies on September 4, 2018^1^. It is difficult as Editor‐in‐Chief of *Journal of Diabetes Investigation* (JDI) to ignore this new challenge. Leaving that topic to be discussed later, it is worth first looking back on JDI's progress over the past ten years.

*J Diabetes Investigation*, the official journal of the Asian Association for the Study of Diabetes (AASD) has reached the 10^th^ Anniversary of its establishment in 2019. In Figure 1, the time course of our journal is shown since its first issue as a bimonthly journal in 2010^3^. Its first Impact Factor was released two years after the launch with a value of 1.861. JDI converted to full Open Access from the first issue in 2014 (Vol. 5, Issue 1)^4^, ^5^, ^6^. At this time, our journal was accepted to PubMed Central and then PubMed/MEDLINE in 2015. As seen in Figure 1, there was a remarkable rise in the Impact Factor following the introduction of Open Access. There was a corresponding increase in the number of downloads, reaching almost 620,000 in 2018. This growth in online usage is expected to continue and judging from figures up to August 2019, it may reach over seven hundred and seventy thousand in 2019. It seems there is a positive relationship between online usage and Impact Factor. Namely, the introduction of Open Access by JDI resulted in the incremental growth of online usage, contributing to the rise in Impact Factor. The 2018 Impact Factor (3.902) of JDI is ranked at 42nd place out of 145 journals in the same subject of Endocrinology and Metabolism.

**Figure 1:**
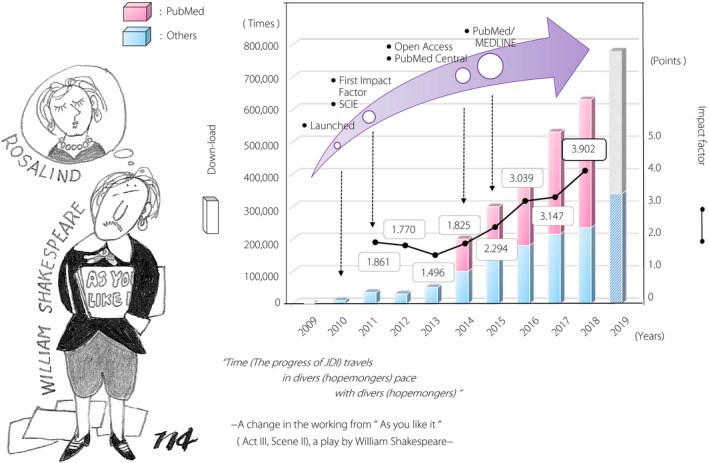
The progress of JDI travels in optimistic paces with optimistic persons. A remarkable rise in Impact Factor was observed after the introduction of Open Access. It seems that there is a positive relationship between online usage and Impact Factor.

At the moment, I am afraid the Impact Factor has the same status for scientific journals as the Michelin Guide does for restaurants. It is true that most medical scientists prefer to submit their fruitful manuscripts to journals with a higher Impact Factor. This is inevitable because the quality of their work is widely evaluated based on Impact Factor. Accordingly, we have no choice but to follow the trends of the time in the world of medical science. Therefore, we will not spare any effort toward our aim to achieve a higher Impact Factor for our team of contributors, readers, reviewers, Editorial Board members and the publisher. We hope that JDI makes a significant contribution to the advancement of medical science and better treatment and care for patients.

Now, let’s turn to ‘Plan S’(https://www.coalition-s.org/). According to an analysis of publishing models in 2017^1^, ^2^, the proportion of journals published in 2016 showed that only 15% were Gold Open Access (where all articles are immediately Open Access on publication^1^); 37.7% were subscription‐only; and 45% were hybrid (that publishes both free and paywalled papers^7^).

This new challenge causes some concerns to us. This program is unlikely to be equivalent between Europe and the United States^8^. Because key US federal agencies such as National Institute of Health (NIH), mandate a ‘green’ Open Access policy, whereby articles in subscription journals are automatically made available after a 12‐month embargo. This policy protects the existing ‘paywalled’ subscription business model.

Also, ‘Plan S’ does not allow for scientists to publish their papers in hybrid journals. Despite an extended timeline for transition away from hybrid journals until 2024^2^ (see https://www.alpsp.org/write/MediaUploads/Reports/SPA%2520OPS/SPA_OPS_final_report.pdf, or https://www.sciencemag.org/news/2019/09/new-deals-could-help-scientific-societies-survive-open-access), the ‘Plan S’ restriction on hybrid journals potentially bars researchers from publishing in 85% of journals, including influential journals like *Nature* and *Science* 1.

One piece of bright news, however, is that Open Access publication fees would be covered by funders or research institutions, not by individual researchers. Moreover, the articles would be free for anyone to download, read or translate without payment.

Given that the publishing industry is approaching a new era in which 85% or more of journals are Open Access, it is necessary for us to develop a survival strategy against this coming fierce competition. Although our journal is already Open Access, we have some concerns regarding the publication fee being covered by either researchers or institutions. No one knows for sure what will happen in the future. It is clear that influential journals, such as *Nature* and *Science*, are unshakable. Our middle‐ranking journals, however, will be forced to make strenuous efforts to preserve high‐quality articles.

For JDI to survive fierce competition, we plan to do the following: (1) give a clear message on the direction of our journal to researchers and readers, (2) shorten the time from first peer review to publication, (3) provide very helpful update papers attractive to many researchers and readers, and (4) differentiate our position from other journals. The reality is that most contributors consider submitting their papers to journals with a higher Impact Factor. We must keep this in mind.

In the future, we will continue to publish new research work and provide new information on diabetes and related matters for researchers and readers. To achieve our aim, we won't hold back our best effort in contributing to the development of diabetology including treatment and care for patients with diabetes. It is natural that we are conscious of our situation as a middle‐ranking journal in the category of Metabolism and Endocrinology and we plan to execute the best policy for our position in the coming new era.

## DISCLOSURE

The author declares no conflict of interest.
